# Acute Pulmonary Oedema and Hypercapnic Respiratory Failure During a Probable Autonomic Dysreflexic Episode in Chronic Cervical Spinal Cord Injury: A Case Report

**DOI:** 10.7759/cureus.119619

**Published:** 2026-11-13

**Authors:** Ameen Ghannoum, Amy-Claire Young, Ranganatha Rao

**Affiliations:** 1 Diabetes and Endocrinology, University Hospitals Coventry and Warwickshire NHS Trust, Coventry, GBR

**Keywords:** acute pulmonary oedema, autonomic dysreflexia, cervical spinal cord injury, hfpef, hypercapnic respiratory failure, tetraplegia

## Abstract

Autonomic dysreflexia is a life-threatening complication affecting individuals with spinal cord injuries at or above the sixth thoracic level. While episodic hypertension is its clinical hallmark, serious cardiovascular and respiratory complications may occur. We report the case of a 67-year-old male with long-standing C5-C6 tetraplegia and established autonomic dysreflexia who was admitted with community-acquired pneumonia (CAP). At approximately 01:00 on hospital day 4, after receiving approximately 3 L of maintenance intravenous fluid for poor oral intake over the preceding 72 hours, he developed sudden severe breathlessness and loss of consciousness despite a previously improving oxygen requirement. Oxygen saturation fell to 77% despite oxygen therapy, while systolic blood pressure rose from a baseline of approximately 125-130 mmHg to 167 mmHg. Physical examination revealed bilateral crackles and reduced air entry. Arterial blood gas analysis showed profound hypercapnic acidaemia (pH 7.009; partial pressure of carbon dioxide 18.5 kPa). A chest radiograph showed rapidly progressive bilateral airspace opacities consistent with pulmonary oedema. His blood pressure returned towards baseline, and following high-concentration oxygen and intravenous furosemide, his oxygen requirement subsequently decreased. Computed tomography pulmonary angiography excluded pulmonary embolism, and echocardiography showed low-normal left ventricular systolic function without a major structural or valvular abnormality. The sudden respiratory deterioration, clinically significant rise in blood pressure from baseline, rapid radiographic evolution, and subsequent improvement supported acute pulmonary oedema occurring during a probable autonomic dysreflexic episode.

No discrete precipitating stimulus was confirmed, although concurrent pneumonia was considered the most likely trigger for autonomic dysreflexia. Recent intravenous fluid administration and underlying cardiac vulnerability may have lowered the threshold for pulmonary oedema.

This case highlights the importance of recognising a clinically meaningful rise from baseline blood pressure when a patient with high spinal cord injury develops sudden respiratory deterioration.

## Introduction

Autonomic dysreflexia is an episodic and life-threatening syndrome seen predominantly after spinal cord injury at or above the sixth thoracic level. It has been reported in 48-91% of people with injuries at or above T6, although estimates vary with injury characteristics and diagnostic criteria [1]. A noxious or non-noxious stimulus below the neurological level of injury can provoke an exaggerated sympathetic response that is no longer adequately regulated by descending supraspinal pathways. This results in widespread vasoconstriction and an abrupt rise in blood pressure. The baroreflex increases vagal activity, which may cause bradycardia, while reduced sympathetic tone causes vasodilatation above the lesion. These responses cannot adequately counter the sympathetic vasoconstriction below it [1,2].

Common triggers include bladder distension, urinary tract pathology, bowel impaction, and skin irritation, although an explicit trigger is not always identified [2]. Resting blood pressure is often low in people with high spinal cord injury, so a rise in systolic pressure of more than 20 mmHg above the individual's baseline may represent clinically important autonomic dysreflexia even if the absolute reading is not striking [1,2]. Typical symptoms include headache, sweating and flushing above the lesion, piloerection, visual disturbance, arrhythmia and anxiety. Severe episodes can lead to seizures, intracranial haemorrhage, myocardial injury or death [1,2].

Pulmonary oedema is a rarely reported complication of autonomic dysreflexia [3,4]. In a review of 32 reported life-threatening complications, only two were pulmonary [5]. A sympathetic surge may cause pressure-driven pulmonary oedema by increasing systemic vascular resistance, left ventricular afterload, central blood redistribution, and pulmonary capillary hydrostatic pressure. Neurogenic pulmonary oedema may also involve increased capillary permeability, and the two mechanisms can overlap [3,4,6]. Cervical spinal cord injury may reduce respiratory muscle reserve and cough effectiveness, increasing vulnerability when acute pulmonary disease develops [7]. We present a case of acute pulmonary oedema and hypercapnic respiratory failure occurring during a probable autonomic dysreflexic episode in a patient with chronic cervical spinal cord injury.

## Case presentation

A 67-year-old male was admitted with a four-day history of increasing breathlessness and a non-productive cough. He denied chest pain, fever, vomiting, or diarrhoea. He had sustained a traumatic C5-C6 spinal cord injury more than three decades previously, resulting in tetraplegia with longstanding sensory impairment below the level of injury. A current neurological level and American Spinal Injury Association Impairment Scale grade were not available. There was no reported recent change in neurological function, and spinal MRI was not performed during this admission. He was wheelchair-dependent, required continuous assistance with daily care, and usually managed his bladder with an external urinary sheath. He had established autonomic dysreflexia, which had previously manifested as episodic hypertension during micturition. His baseline systolic blood pressure was typically 125-130 mmHg. He had no previous diagnosis of hypertension and was not taking regular antihypertensive medication before admission.

During the admission, he had intermittent rises in systolic blood pressure of up to 189 mmHg, consistent with his known episodic dysreflexia. Immediately before the acute deterioration, his systolic blood pressure had returned to approximately 125-130 mmHg. Oxygen saturation on admission was 96% on 4 L/min via nasal cannulae. An admission arterial blood gas on this oxygen therapy showed a pH of 7.31, arterial partial pressure of oxygen (PaO_2_) of 10.9 kPa, arterial partial pressure of carbon dioxide (PaCO_2_) of 5.96 kPa, standard bicarbonate of 21.7 mmol/L, and base excess of -3.1 mmol/L. He did not use home oxygen and had no established chronic respiratory failure. Laboratory investigations revealed a white cell count of 12 × 10⁹/L, C-reactive protein of 112 mg/L, B-type natriuretic peptide of 6,359 ng/L, urea of 11 mmol/L, and creatinine of 86 µmol/L. The initial chest radiograph demonstrated patchy bilateral airspace changes.

He was started on intravenous co-amoxiclav, corticosteroids, and nebulised bronchodilators for community-acquired pneumonia (CAP). No wheeze was documented on admission or during the subsequent deterioration, and no response to salbutamol was recorded. Over the following 72 hours, he received approximately 3 L of maintenance intravenous fluid because of poor oral intake. A urethral catheter inserted on hospital day 2 was documented to be draining adequately during the subsequent deterioration. He had an unstageable sacral pressure ulcer and category 2 pressure ulcers over both buttocks, which were dressed daily and remained dry. Previous infection of a pressure ulcer had triggered autonomic dysreflexia. His oxygen requirement decreased to 3 L/min, with stable respiratory parameters.

At approximately 01:00 on hospital day 4, following a period of relative clinical stability (respiratory rate 17 breaths/min, oxygen saturation 96% on 3 L/min), he developed sudden severe dyspnoea, laboured breathing, and rapid deterioration of consciousness. Oxygen saturation dropped to 77% on 3 L/min oxygen, and his respiratory rate increased to 26 breaths/min. During the deterioration, his blood pressure was 164/84 mmHg, with a subsequent systolic reading of 167 mmHg, representing a rise of more than 35 mmHg above his immediately preceding baseline. His heart rate was 101 beats/min. He became completely unresponsive. Chest auscultation revealed widespread bilateral crackles and reduced air entry.

A nasopharyngeal airway was inserted, and oxygen was increased to 15 L/min via a non-rebreather mask, raising his saturation to 90%. An arterial blood gas obtained during the acute deterioration at approximately 01:07, while he was receiving this treatment, showed profound hypercapnic acidaemia. The exact delivered fraction of inspired oxygen (FiO_2_) was not measured, so a reliable PaO_2_/FiO_2_ ratio could not be calculated (Table 1).

**Table 1:** Arterial blood gas results during acute deterioration The sample was obtained on 15 L/min oxygen via a non-rebreather mask; FiO₂ was not measured. The PaO₂ reference range assumes room air. Reported HCO₃⁻ represents standard bicarbonate; actual bicarbonate was unavailable. PaCO₂: arterial partial pressure of carbon dioxide; PaO₂: arterial partial pressure of oxygen; HCO₃⁻: bicarbonate.

Parameter	Result	Reference range	Unit
pH	7.009	7.35-7.45	--
PaCO₂	18.5	4.7-6.0	kPa
PaO₂	10.2	10.0-13.3	kPa
HCO₃⁻ (standard)	21.3	22-26	mmol/L
Lactate	1.0	0.5-1.6	mmol/L

Hydrocortisone 100 mg and nebulised salbutamol were administered. Morphine 2.5 mg and midazolam 5 mg were given as anticipatory medication approximately 90 minutes after the deterioration began, so they could not account for the initial collapse. Naloxone given later produced no clinical improvement. No ECG or troponin measurement was obtained during the acute event. During the deterioration, a ward-based ceiling of care was agreed, and he was considered unsuitable for non-invasive ventilation, intensive care admission, or invasive mechanical ventilation.

A portable chest radiograph obtained within 1 hour of deterioration demonstrated extensive bilateral consolidation and airspace shadowing that had rapidly progressed compared with the admission image. The acute clinical and radiographic deterioration was considered consistent with acute pulmonary oedema occurring during a probable autonomic dysreflexic episode. No discrete precipitating stimulus was confirmed. The urinary catheter was draining adequately, making substantial bladder distension from complete obstruction less likely, although a urinary trigger could not be excluded. The pressure ulcers remained dry without evidence of active infection. Bowel status, restrictive clothing, positioning, and other potential triggers were not systematically documented because autonomic dysreflexia was not suspected during the emergency. Concurrent pneumonia was considered the most likely trigger.

He was initially unresponsive and later became conscious but agitated. At approximately 10:00, he was asleep when reviewed; interpretation of his level of consciousness was complicated by the anticipatory morphine and midazolam given after the initial deterioration. His blood pressure had fallen to 139/88 mmHg by 10:53, before intravenous furosemide 40 mg was administered at 11:22, and was 111/62 mmHg at 12:24. His oxygen requirement decreased from 15 L/min to 4 L/min by 14:13. By approximately 01:00 the following day, he was fully alert, comfortable, and speaking in complete sentences. Intravenous furosemide was continued daily before being changed to oral treatment. Serial chest radiographs showed the rapid deterioration and subsequent improvement (Figure 1).

**Figure 1:**
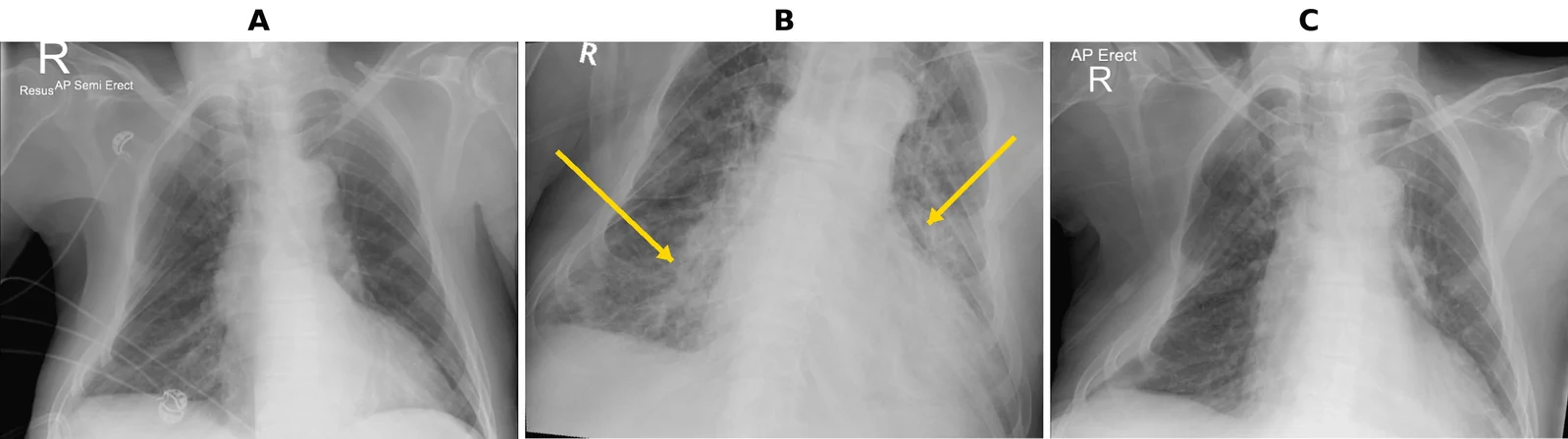
Serial portable anteroposterior chest radiographs Serial anteroposterior chest radiographs obtained: (A) on admission at 09:09 in the semi-erect position; (B) during acute deterioration on hospital day 4 at 01:43 using a portable machine; and (C) six days after the event at 11:43 in the erect position. Panel B shows rapid progression of bilateral airspace opacification, greater on the left, with the principal areas of increased shadowing marked by yellow arrows. The left-sided predominance may reflect concurrent left lower lobe pneumonia and pleural effusion. Panel C shows substantial interval improvement. AP: anteroposterior.

Computed tomography pulmonary angiography excluded pulmonary embolism and showed left lower lobe consolidation with a pleural effusion and right lower lobe atelectasis. Transthoracic echocardiography four days after the event showed concentric left ventricular hypertrophy and left atrial enlargement. Septal and lateral e′ velocities were reduced at 3.8 and 6.5 cm/s, with E/e′ ratios of 22.0 and 12.9, respectively. These findings supported impaired diastolic function and elevated left ventricular filling pressure. Left ventricular systolic function was low-normal, with an estimated ejection fraction of approximately 50%. There was no major valvular abnormality sufficient to explain the abrupt deterioration. Cardiology considered the findings consistent with heart failure with preserved ejection fraction.

New-onset atrial fibrillation was detected on day 12 and managed with apixaban. He was successfully weaned off oxygen and discharged on day 35 with oral furosemide, dapagliflozin, and apixaban. Glyceryl trinitrate (GTN) spray was prescribed on discharge for future episodes of autonomic dysreflexia. He and his carers were provided with written resources on recognising and managing autonomic dysreflexia. These included monitoring for a rise in blood pressure from his usual baseline, identifying and addressing common triggers, particularly bladder or bowel disturbance, and using GTN spray if systolic blood pressure exceeded 150 mmHg.

## Discussion

The rise in systolic blood pressure of more than 35 mmHg from the immediately preceding baseline met the recognised haemodynamic criterion for autonomic dysreflexia in a patient with chronic high-level spinal cord injury and established dysreflexic hypertension [1,2]. Classic symptoms above the lesion were not documented, and it is unclear whether they were specifically assessed, as autonomic dysreflexia was not suspected during the emergency. His rapid loss of consciousness also limited symptom reporting, and autonomic dysreflexia can be asymptomatic [1,2]. Tachycardia does not exclude autonomic dysreflexia during infection or severe physiological stress [1,2]. No single trigger was confirmed. Pneumonia was considered the most likely trigger. A urinary trigger could not be excluded despite adequate catheter drainage, while the pressure ulcers remained dry without evidence of active infection. No autonomic dysreflexia-specific antihypertensive was administered. Hypoxaemia, hypercapnia, and respiratory distress could also have contributed to the rise in blood pressure. Overall, the findings supported a probable autonomic dysreflexic episode but did not establish it as the sole cause of pulmonary oedema.

Several features supported acute pulmonary oedema rather than uncomplicated progression of pneumonia alone. His respiratory status and oxygen requirement had initially improved before the sudden onset of severe hypoxaemia, bilateral crackles, reduced air entry, and loss of consciousness. This deterioration coincided with a clinically meaningful rise in blood pressure and rapid progression of bilateral airspace shadowing. His blood pressure returned towards baseline before intravenous furosemide was administered, whereas his oxygen requirement decreased after diuresis later that day. Exclusion of pulmonary embolism, subsequent radiographic improvement, and the absence of a major structural or valvular cardiac abnormality supported an acute pulmonary oedematous component.

Previous case reports described pulmonary oedema associated with autonomic dysreflexia as acute neurogenic pulmonary oedema [3,4]. In this setting, neurogenic describes the likely sympathetic trigger, while flash describes the rapid onset. Neurogenic pulmonary oedema can result from hydrostatic, permeability-mediated, or mixed mechanisms [6]. In this case, the rise in blood pressure, underlying cardiac vulnerability, recent intravenous fluids, and response to diuresis pointed to a mainly haemodynamic mechanism, with increased left ventricular afterload and pulmonary capillary hydrostatic pressure. Pulmonary capillary permeability was not measured, and other cardiopulmonary contributors were present. The event is therefore described as acute pulmonary oedema occurring during a probable autonomic dysreflexic episode. Key clinical features of the two published cases and the present case are compared in Table 2 [3-5].

**Table 2:** Comparison with published cases of pulmonary oedema associated with autonomic dysreflexia Published case data were drawn from the original reports and a later clinical review [3-5]. AD: autonomic dysreflexia; AIS: American Spinal Injury Association Impairment Scale; BP: blood pressure; HR: heart rate; RR: respiratory rate; SBP: systolic blood pressure.

Feature	Kiker et al. (1982) [4]	Calder et al. (2009) [3]	Present case
Patient and spinal injury	32-year-old man; C6-C7 spinal cord injury	50-year-old man; chronic C5 AIS B injury for 22 years	67-year-old man; chronic C5-C6 injury with tetraplegia for more than three decades
Trigger or clinical context	External sphincterotomy; traction from an over-inflated Foley catheter balloon	Bowel preparation, constipation and bowel movements	Pneumonia considered the most likely trigger; no single stimulus confirmed; recent intravenous fluids and diastolic dysfunction were contributory
Blood pressure and heart rate	BP 162/110-170/118 mmHg; HR 88-100 beats/min	First AD episode: BP 210/110 mmHg. Respiratory event: BP 154/111 mmHg; HR 160 beats/min	Baseline SBP 125-130 mmHg; event BP 164/84 mmHg followed by SBP 167 mmHg; HR 101 beats/min
Respiratory presentation	Pulmonary oedema after intense, prolonged AD	Acute dyspnoea; RR 38 breaths/min; oxygen saturation 73% on room air; bilateral infiltrates	Sudden dyspnoea and unresponsiveness; oxygen saturation 77% on 3 L/min; severe hypercapnic acidaemia; rapidly progressive bilateral opacification
Management	Foley balloon deflated, removing the trigger	Oral and topical antihypertensives; intensive care transfer and intubation; resuscitation after asystole	Nasopharyngeal airway, 15 L/min oxygen through a non-rebreather mask and intravenous furosemide; ward-based ceiling of care
Outcome	Prompt resolution of autonomic dysreflexia and pulmonary oedema	Died on hospital day 4 after cardiac arrest and subsequent conservative management	Blood pressure returned towards baseline; oxygen requirement improved after diuresis later that day; fully alert by approximately 24 hours; discharged on hospital day 35

The presentation was likely multifactorial. Earlier hypertensive episodes had not caused pulmonary oedema, suggesting that autonomic dysreflexia alone was insufficient. Infection, recent intravenous fluids, and underlying cardiac vulnerability may have lowered the threshold for the later event. Fluid overload or decompensated heart failure was considered because he had received intravenous fluids, had an elevated B-type natriuretic peptide, and was later diagnosed with heart failure with preserved ejection fraction. However, he had no previous history of heart failure, peripheral oedema, or raised jugular venous pressure, and his oxygen requirement had been improving before the event. Prednisolone may have contributed to fluid retention, but hydrocortisone given during the deterioration could not explain its onset. Pneumonia with an effusion remained a contributor and a possible trigger for autonomic dysreflexia.

No aspiration event or mucus plugging was documented. Right lower lobe atelectasis may have impaired gas exchange but did not explain the rapid bilateral changes. The greater left-sided shadowing could reflect left lower lobe infection superimposed on pulmonary oedema. Opioid-induced hypoventilation was unlikely to explain the onset because morphine and midazolam were given after the deterioration, and naloxone produced no improvement. No single alternative diagnosis fully accounted for all features, although several likely contributed.

One possible sequence is that autonomic dysreflexia caused a sympathetic surge and increased left ventricular afterload in a patient already vulnerable to pulmonary congestion because of pneumonia, recent intravenous fluids, and previously unrecognised diastolic dysfunction. This may have led to rapid pulmonary fluid accumulation followed by hypoxaemic and hypercapnic respiratory failure. Respiratory muscle fatigue and alveolar hypoventilation may then have worsened carbon dioxide retention, with hypercapnic acidaemia contributing to the loss of consciousness [7].

Other mechanisms may also have contributed. Autonomic dysreflexia-associated vagal bronchoconstriction can cause hypercapnic respiratory failure [8], although no wheeze or clear response to salbutamol was documented. Oxygen-associated hypercapnia was unlikely to explain the initial deterioration because he had been receiving 3 L/min oxygen and had no established chronic respiratory disease or home oxygen requirement. The admission blood gas did not show an elevated standard bicarbonate to suggest established metabolic compensation for chronic carbon dioxide retention. The arterial blood gas during the event was obtained after oxygen was increased to 15 L/min. Alternatively, decompensated heart failure with preserved ejection fraction after fluid administration could have been the main driver, with the rise in blood pressure occurring partly in response to respiratory distress. The available retrospective data cannot establish the exact sequence or contribution of each process. The proposed multifactorial pathway is shown in Figure 2.

**Figure 2:**
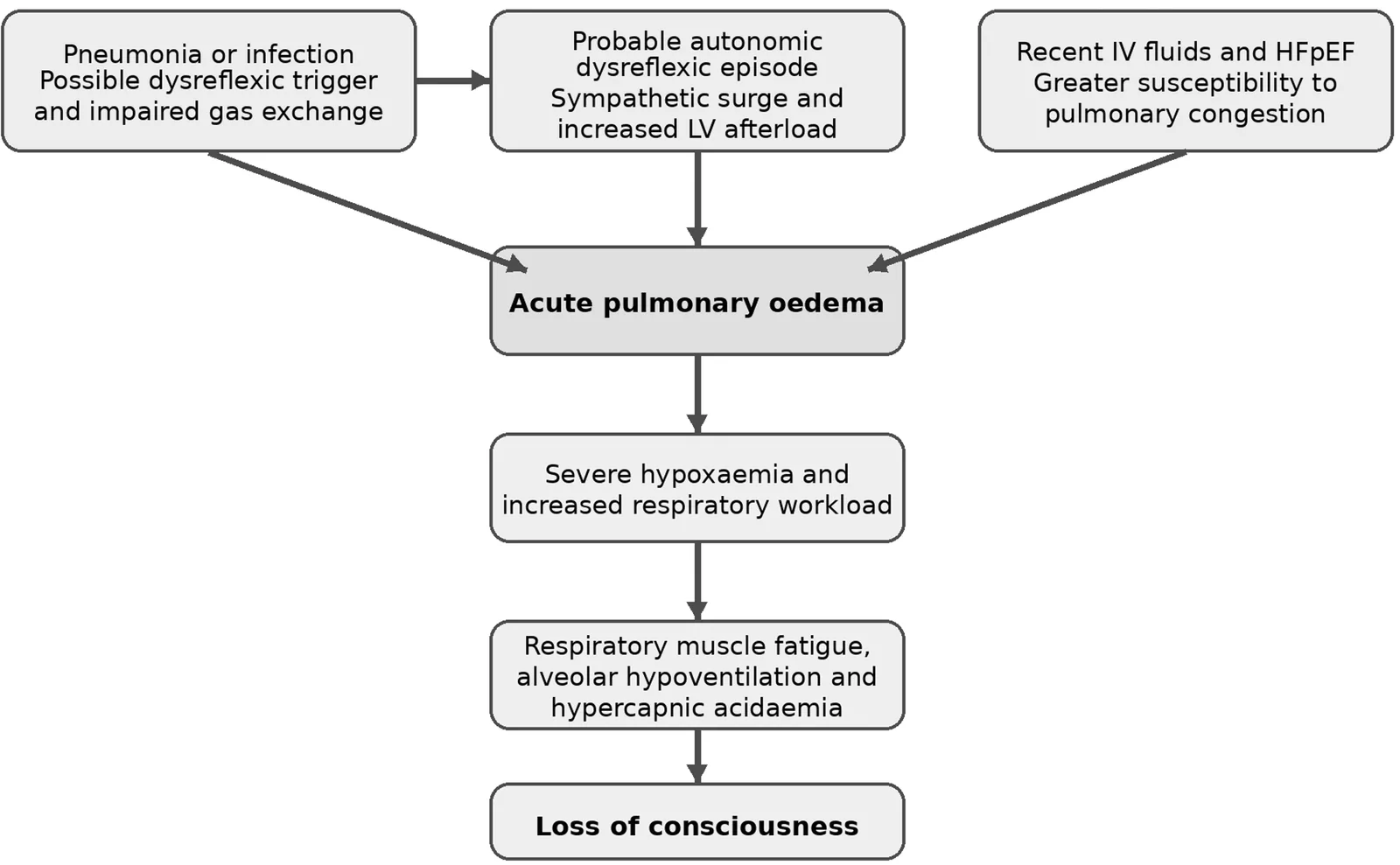
Proposed multifactorial pathway Pneumonia, probable autonomic dysreflexia, IV fluids, and diastolic dysfunction may have contributed to acute pulmonary oedema and hypercapnic respiratory failure; their relative timing and contributions remain uncertain. HFpEF: heart failure with preserved ejection fraction; IV: intravenous; LV: left ventricular.

Autonomic dysreflexia is a medical emergency. Management requires prompt recognition, frequent blood pressure monitoring, removal of possible triggers, and a short-acting antihypertensive when indicated [2,9]. If pulmonary oedema or hypercapnic respiratory failure develops, autonomic dysreflexia and the cardiopulmonary complication should be treated at the same time (Table 3) [2,7,9,10].

**Table 3:** Acute management of autonomic dysreflexia and associated cardiopulmonary complications Management recommendations are summarised from published guidance on autonomic dysreflexia, respiratory failure, and acute pulmonary oedema [2,7,9,10]. GTN: glyceryl trinitrate; PDE5: phosphodiesterase type 5.

Clinical scenario	Management
Suspected autonomic dysreflexia	Sit the patient upright, loosen restrictive clothing, and compare systolic blood pressure with the usual baseline.
Monitoring	Record blood pressure and pulse every 2-5 minutes until stable.
Trigger search	Check the bladder and catheter drainage first, then assess the bowel, skin and pressure areas, pain, and infection.
Persistent hypertension	If systolic blood pressure remains at or above 150 mmHg, use a rapid-onset, short-duration agent according to local guidance, such as GTN spray or immediate-release nifedipine. Check for recent PDE5-inhibitor use before GTN and monitor for hypotension.
After resolution	Monitor for recurrent hypertension or treatment-related hypotension. Provide an individualised trigger plan and prescribed rescue medication.
Pulmonary oedema	Support the airway, give oxygen, reassess the need for ongoing intravenous fluids, give an intravenous loop diuretic, and monitor renal function and urine output.
Hypercapnic respiratory failure	Consider non-invasive ventilation for severe dyspnoea with acidaemia when clinically appropriate and within the agreed ceiling of care.

In this case, the immediate priority was acute respiratory failure with suspected pulmonary oedema. Airway support and high-concentration oxygen were provided within the agreed ward-based ceiling of care. His blood pressure returned towards baseline before intravenous furosemide was administered, while his oxygen requirement decreased later that day after diuresis. By approximately 24 hours after the event, he was fully alert and comfortable on 4 L/min oxygen. Autonomic dysreflexia was not suspected during the initial emergency response and was recognised retrospectively as a probable contributor after the clinical course was reviewed. A short-acting nitrate was therefore not given during the event.

Limited physician awareness may contribute to delayed recognition of autonomic dysreflexia. In a questionnaire study of 95 physicians from specialties likely to encounter the condition, 40% could not answer any questions about autonomic dysreflexia, only 27% had received relevant training, and 18% had previous clinical experience [11]. The retrospective recognition in this case highlights the need for acute medical teams to consider autonomic dysreflexia early when a patient with high-level spinal cord injury deteriorates, including when respiratory failure or pulmonary oedema dominates the presentation.

This report has several limitations. Autonomic dysreflexia was identified retrospectively, no single trigger was confirmed, and typical symptoms above the level of injury were neither documented nor known to have been systematically assessed. Pneumonia, maintenance intravenous fluids, and previously unrecognised diastolic dysfunction may all have contributed. A complete fluid balance and quantitative urine output were unavailable, although the urinary catheter was documented to be draining. The exact inspired oxygen concentration, ECG, and troponin measurements were also unavailable during the event. The temporal association supports autonomic dysreflexia as a probable contributor, but the available data cannot establish whether the rise in blood pressure preceded or partly resulted from the respiratory deterioration, or prove that autonomic dysreflexia was the sole cause of pulmonary oedema.

## Conclusions

Acute pulmonary oedema is a rare but life-threatening event that may occur in association with autonomic dysreflexia in chronic high-level spinal cord injury. When a patient with tetraplegia deteriorates suddenly, a clinically meaningful rise in blood pressure from the individual's baseline should prompt a systematic search for dysreflexic triggers while concurrent causes, including infection, fluid overload, cardiac dysfunction, aspiration, and medication-related hypoventilation, are considered. Ongoing intravenous fluid requirements and cumulative fluid balance should be reassessed frequently, especially when infection or diastolic dysfunction may increase susceptibility to pulmonary congestion. Greater awareness among acute medical teams may support earlier recognition and treatment of autonomic dysreflexia alongside prompt respiratory support and management of pulmonary oedema.
